# The Capio Prostate Cancer Center Model for Prostate Cancer Diagnostics—Real-world Evidence from 2018 to 2022

**DOI:** 10.1016/j.euros.2024.01.012

**Published:** 2024-02-06

**Authors:** Thorgerdur Palsdottir, Harald Söderbäck, Fredrik Jäderling, Martin Bergman, Hari Vigneswaran, Henrik Grönberg

**Affiliations:** a Department of Medical Epidemiology and Biostatistics, Karolinska Institutet, Solna, Sweden; b Department of Oncology, Capio St. Görans Sjukhus, Stockholm, Sweden; c Urokirurgist Centrum, Capio, Stockholm, Sweden

**Keywords:** Prostate cancer, Risk prediction, Prostate cancer risk, Prostate-specific antigen, Stockholm3, Prostate cancer diagnostics, Magnetic resonance imaging

## Abstract

**Background:**

The Capio Prostate Cancer Center (Capio PCC) in Stockholm, Sweden, adopts a comprehensive diagnostic approach, utilizing prostate-specific antigen (PSA), Stockholm3, and magnetic resonance imaging (MRI) for prostate cancer risk assessment, followed by targeted and systematic biopsies for high-risk cases.

**Objective:**

This study aims to elucidate the clinical process and real-world outcomes of the Capio PCC model for prostate cancer diagnosis at Capio S:t Göran Hospital.

**Design, setting, and participants:**

Between 2018 and 2022, a cohort of 12 406 men aged 45–75 yr underwent prostate cancer testing, adhering to Capio PCC's structured diagnostic protocol.

**Outcome measurements and statistical analysis:**

We provide a comprehensive description of the Capio PCC model and present results from its implementation, including assessments of PSA, Stockholm3, MRI scans, and biopsies. A comparative analysis is conducted between the diagnostic outcomes obtained at Capio PCC and those obtained at other regions in Sweden.

**Results and limitations:**

The median participant age was 61 yr (interquartile range [IQR]: 55–67), with PSA levels at 1.6 ng/ml (IQR: 0.8–3.3) and Stockholm3 scores at 4 (IQR: 3–11). Among 1064 men (8.6%) undergoing biopsies, 611 (57% of biopsied) were diagnosed with International Society of Urological Pathology grade ≥ 2 cancer. Notably, employing a Stockholm3 ≥ 15 cutoff for biopsy, in lieu of PSA ≥ 3 ng/ml, reduced biopsy recommendations by 43%. For men with PSA levels between 1.5 and 2.9 ng/ml, 360 (12%) exhibited Stockholm3 scores of ≥ 15, with 72 (56% of biopsied) diagnosed with clinically significant prostate cancer. A comparative analysis with national Swedish prostate cancer detection data indicated that the Capio PCC model (vs Sweden) revealed a distribution of 14% (vs 25%) low-risk, 59% (vs 42%) intermediate-risk, and 26% (vs 30%) high-risk and advanced cancers.

**Conclusions:**

This study underscores the effectiveness of the protocol-driven diagnostic process at Capio PCC, enabling earlier detection of intermediate-risk prostate cancer and reducing the need for MRI assessments compared with standard prostate cancer care in Sweden.

**Patient summary:**

At the Capio Prostate Cancer Center, a novel diagnostic approach incorporating prostate-specific antigen, Stockholm3, magnetic resonance imaging, and targeted biopsies has been implemented to enhance prostate cancer testing and diagnosis in Stockholm, Sweden.

## Take Home Message

At the Capio Prostate Cancer Center, a new diagnostic process using prostate-specific antigen, Stockholm3, magnetic resonance imaging, and targeted biopsies has been implemented to improve prostate cancer testing and diagnosis in Stockholm, Sweden.

## 1 Introduction

Population-based screening studies using the prostate-specific antigen (PSA) test and systematic biopsies have indicated a reduction in prostate cancer mortality [1], [2]. However, PSA testing, although recommended with shared decision-making, has limitations due to overdiagnosis and associated treatment side effects [3], [4]. In response, the Capio S:t Göran Hospital in Stockholm, Sweden, established the Capio S:t Göran Prostate Cancer Center (Capio PCC) in 2017 [5], [6]. Capio PCC introduced a protocol-driven diagnostic process featuring the blood-based diagnostic test Stockholm3, magnetic resonance imaging (MRI), and targeted and systematic biopsies, delegating many responsibilities from medical doctors to specially trained nurses and assistant nurses.

Several blood-based tests, when combined with systematic and targeted biopsies, show potential for reducing overdiagnosis and overtreatment [7], [8], [9], [10]. One such test is Stockholm3, which incorporates clinical variables, biomarkers, and a polygenic risk score to predict clinically significant prostate cancer [7]. Stockholm3 research suggests a potential reduction of up to 50% in unnecessary biopsies, a 35% decrease in MRI usage, and a 20–30% decrease in low-risk prostate cancer diagnoses, while maintaining the detection rates comparable with the traditional PSA tests for clinically significant prostate cancer [7], [10], [11].

Additionally, MRI plays a vital role in prostate cancer screening, guiding biopsies toward suspicious lesions and reducing unnecessary procedures [12], [13], [14], [15]. Combining Stockholm3's improved risk prediction with MRI-targeted biopsies could improve the efficacy of prostate cancer screening. The 2021 STHLM3-MRI screening trial reported a 67% reduction in the detection of clinically nonsignificant prostate cancer and a 17% increase in detection of clinically significant cases compared with PSA and systematic biopsy [10]. Nordström et al [10] also demonstrated that integration of Stockholm3 and MRI improved the sensitivity of the test compared with the original study and, by increasing the Stockholm3 threshold to 15, achieved similar sensitivity to PSA ≥ 3 ng/ml for detecting clinically significant prostate cancer.

The Capio PCC diagnostic process is evidence based, and this study aims to describe the process, present real-world results from 2018 to 2022, and compare detection of clinically significant prostate cancer at Capio PCC to national prostate cancer data in Sweden.

## 2 Patients and methods

### 2.1 The Capio PCC model for prostate cancer diagnosis

**Fig. 1:**
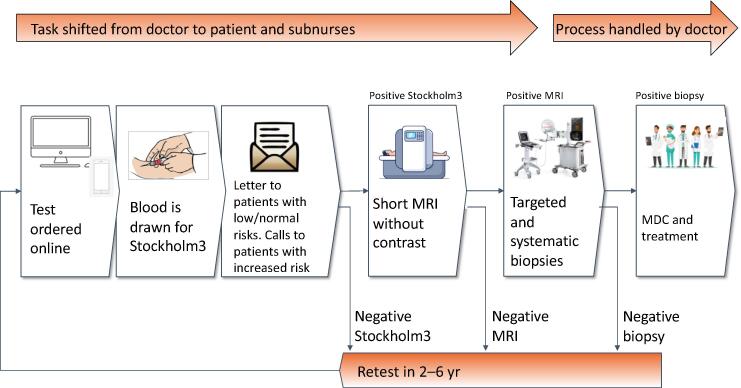
Standardized diagnostic process at Capio PCC. Capio PCC = Capio Prostate Cancer Center; MDC = multidisciplinary conference; MRI = magnetic resonance imaging.

The Capio S:t Göran Hospital is located in central Stockholm, and in 2017, Capio PCC was established with an aim to provide improved and cost-effective prostate cancer care in Stockholm with shortened waiting times for patients. It is mandated by Stockholm County Council to handle approximately 25% of prostate cancer care in Stockholm and aim to enhance the diagnosis and treatment of prostate cancer in Stockholm using contemporary diagnostic techniques. Furthermore, a protocol-driven and efficient process was implemented, delegating a substantial proportion of responsibilities from medical doctors to trained assistant nurses and integrating up-to-date clinical research. Known as the Capio PCC model, the diagnostic chain includes PSA testing, Stockholm3 test, and MRI, followed by targeted and systematic biopsies (see Fig. 1) [5], [6], [7], [10], [11], [12]. The Stockholm3 test is a multiplex blood-based test that predicts the risk of clinically significant prostate cancer (defined as International Society of Urological Pathology [ISUP] grade ≥ 2 on biopsy). The test combines protein biomarkers, clinical variables, and a genetic risk score in a risk prediction model, and results in recommendation for MRI or prostate biopsy.

More precisely, the process at Capio PCC starts when men requesting testing (self-referral) or referred from general practitioners complete an online questionnaire to determine eligibility for testing. Men aged 45–75 yr without a previous prostate cancer diagnosis are considered eligible for testing. The questionnaire gathers information on PSA testing history, disease history, family history of prostate cancer, and previous negative biopsies or MRI (questionnaire is available in the Supplementary material). Eligible men undergo a blood test and those at an increased risk on blood test (men with PSA ≥ 1.5 ng/ml are additionally tested with Stockholm3 and those with Stockholm3 ≥ 15 are at an increased risk) undergo MRI, and based on the results, targeted and systematic biopsies are conducted in men with Prostate Imaging Reporting and Data System (PI-RADS) scores ≥ 3 (Table 1) [16]. Every MRI scan is reviewed by two radiologists, and positive MRI scans are reviewed in a multidisciplinary conference. Furthermore, all positive biopsies are evaluated in a multidisciplinary conference to plan patient treatment. The Capio PCC model minimizes urologist visits, employing dedicated nurses and assistant nurses for test coordination and result communication.

**Table 1:** Study cohort characteristics for men tested with Stockholm3 at Capio PCC in 2018–2022

	2018–2022		2018–2021(Stockholm3 ≥ 11)		2022 (Stockholm3 ≥ 15)	
	Tested men	Biopsied men	Tested men	Biopsied men	Tested men	Biopsied men
	*N* = 12 406	*N* = 1064	*N* = 5439	*N* = 744	*N* = 6967	*N* = 322
Age (yr), median (IQR)	61 (55–67)	66 (61–70)	62 (56–68)	66 (61–70)	60 (54–66)	66 (61–71)
Stockholm3 score a (risk score), median (IQR)	4 (3–11)	22 (15–33)	5 (3–12)	21 (14–33)	3 (3–10)	23 (16–33)
Stockholm3 ≥ 11, *n* (% of total)	–	–	1560 (29)	692 (93)	–	–
Stockholm3 ≥ 15, *n* (% of total)	2002 (16)	809 (75)	1036 (19)	523 (70)	966 (14)	286 (89)
PSA (ng/ml), median (IQR)	1.6 (0.8–3.3)	4.7 (3.3–7.5)	2 (0.9–3.9)	4.5 (3.2–7.4)	1.4 (0.8–2.8)	4.8 (3.4–7.7)
PSA ≥ 3 ng/ml, *n* (% of total)	3496 (28)	852 (80)	1922 (35)	589 (79)	1574 (23)	263 (82)
Prostate volume (ml), median (IQR)	–	40 (30–55)	–	40 (30–55)	–	41 (30–55)
PI-RADS score, *N* (% column)						
3	–	432 (41)	–	325 (44)	–	113 (35)
4	–	343 (32)	–	215 (29)	–	130 (40)
5	–	172 (16)	–	123 (17)	–	54 (17)
No MRI done, *N* (% column)	–	29 (3)	–	20 (3)	–	9 (1)
Biopsy results, *N* (% column)						
Benign	–	332 (31)	–	242 (33)	–	90 (28)
ISUP 1	–	121 (11)	–	88 (12)	–	32 (10)
ISUP 2	–	434 (41)	–	302 (41)	–	134 (42)
ISUP ≥ 3	–	177 (17)	–	112 (15)	–	66 (20)

IQR = interquartile range; ISUP = International Society of Urological Pathology; MRI = magnetic resonance imaging; PI-RADS = Prostate Imaging Reporting and Data System; PSA = prostate-specific antigen.

a This number includes all PSA-tested men in our cohort; while only men with PSA ≥ 1.5 ng/ml undergo Stockholm3 testing, men with PSA  < 1.5 ng/ml will automatically receive a Stockholm3 risk score of 3.

### 2.2 Statistical analysis

Clinical data were gathered systematically from the Stockholm PSA and Biopsy Registry for men between the ages of 45 and 75 yr who underwent Stockholm3 testing between 2018 and 2022. We excluded individuals lacking a Stockholm3 score or a PSA test (a Consort diagram is available in the Supplementary material). We conducted a comprehensive comparison between Stockholm3 and PSA, and collected all relevant MRI and biopsy data for the included individuals.

To gauge the risk at the time of diagnosis, we drew upon regional data from the National Prostate Cancer Registry of Sweden [17]. This involved categorizing men aged 45–75 yr diagnosed with prostate cancer in Sweden from 2018 to 2022 based on their prognosis and available treatment options. The risk categories were defined as follows: low risk: T1–2, ISUP grade 1, and PSA < 10 ng/ml; intermediate risk: T1–2, ISUP grade 2–3, and/or 10 ≤ PSA < 20 ng/ml; high risk: T3 and/or ISUP grade 4–5, and/or 20 ≤ PSA < 50 ng/ml; locally advanced: T4 and/or N1, and/or 50 ≤ PSA < 100 ng/ml; and metastasis: M1 and/or PSA ≥ 100 ng/ml. We meticulously compiled data on individuals diagnosed at Capio PCC and compared these with national and regional data from Sweden.

In addition, we conducted a health economic assessment by comparing the costs associated with the Capio PCC approach to the current standard of care in Sweden. The health economic analysis was based on assumptions rooted in the Swedish healthcare system, with the assumptions detailed in Supplementary Figure 4.

For all our statistical analyses, we employed the R statistical software version 4.2.3.

## 3 Results

Between May 2018 and December 2022, data were collected for 12 406 men undergoing prostate cancer testing at Capio PCC. The median age was 61 yr (interquartile range [IQR]: 55–67), with the median Stockholm3 and PSA levels of 4 (IQR: 3–11) and 1.6 ng/ml (IQR: 0.8–3.3), respectively. Biopsied men had a median age of 66 yr (IQR: 61–70), median Stockholm3 of 22 (IQR: 15–33%), and median PSA of 4.7 ng/ml (IQR: 3.3–7.5).

Up to 2021, a Stockholm3 threshold of 11 was used to indicate MRI. Following the STHLM3-MRI study's findings, the threshold was raised to 15 in January 2022.

**Fig. 2:**
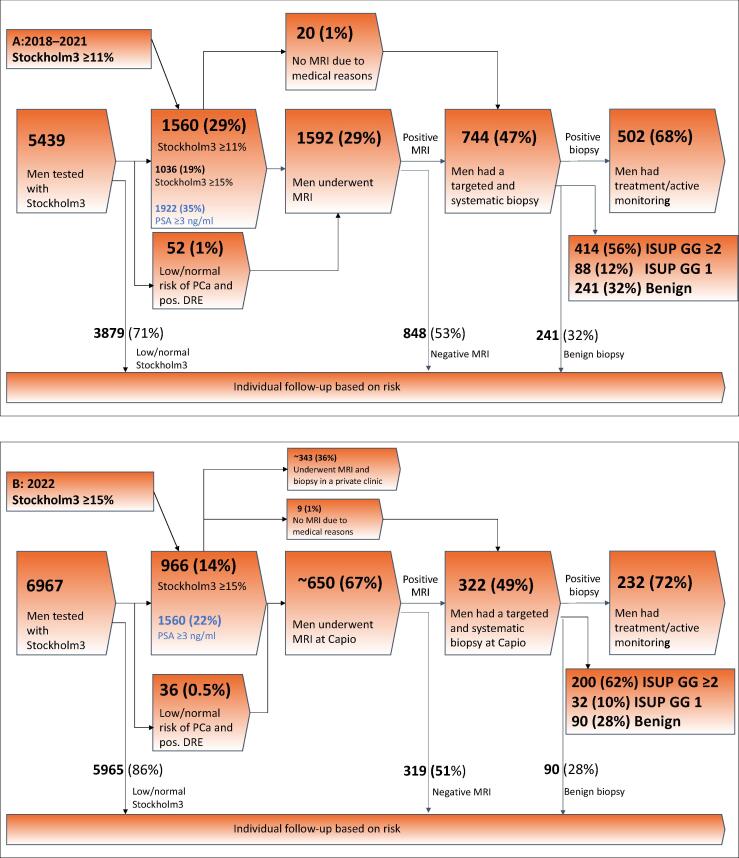
(A) Real-world evidence from Capio PCC for men who followed the clinical process from 2018 to 2021: numbers of men tested with Stockholm3, men undergoing MRI and biopsy, as well as men diagnosed with prostate cancer using a Stockholm3 threshold of 11. (B) Real-world evidence from Capio S:t Göran for men who followed the clinical process in 2022: numbers of men tested with Stockholm3, men undergoing MRI and biopsy, as well as men diagnosed with prostate cancer using a Stockholm3 threshold of 15. Capio PCC = Capio Prostate Cancer Center; DRE = digital rectal examination; GG = grade group; ISUP = International Society of Urological Pathology; MRI = magnetic resonance imaging; PCa = prostate cancer; pos. = positive.

Figure 2A shows that between 2018 and 2021, 5439 men were tested for prostate cancer, and of them, 1560 (29%) had Stockholm ≥ 11, 1036 (19%) had Stockholm3 ≥ 15, and 1922 (35%) had PSA ≥ 3 ng/ml. Subsequently, 1592 men underwent MRI (29%), and 744 (47%) had positive MRI results (PI-RADS score ≥ 3) and underwent a targeted and systematic biopsy. Of the biopsied men, 414 (56%) were diagnosed with clinically significant prostate cancer, 88 (12%) were diagnosed with clinically nonsignificant prostate cancer (ISUP grade 1), and 242 (33%) had a benign biopsy. Compared with using PSA ≥ 3 ng/ml as an indication to MRI, using Stockholm3 with a threshold of 15 decreased the number of those with an increased risk by 46% (from 35% of the tested men to 19%).

In 2022 (Fig. 2B), 966 (14%) men had an increased risk using the Stockholm3 threshold of 15, compared with 1560 (22%) men with PSA ≥ 3 ng/ml. Of the men with an elevated Stockholm3 risk, ∼650 underwent MRI at Capio S:t Göran, while ∼340 underwent MRI at other outpatient radiology departments or urology clinics. Among them, 322 (49%) had positive results and were sent for a biopsy, with 200 men (62%) diagnosed with clinically significant prostate cancer, 32 (10%) diagnosed with clinically nonsignificant prostate cancer, and 90 (28%) having a benign biopsy. Using Stockholm3 compared with PSA ≥ 3 ng/ml reduced the number of individuals identified to have a high risk by 36%, from 22% to 14%. Among the men who had negative or normal recommendation of Stockholm3 but a PSA value of ≥ 3 ng/ml, healthcare workers followed the result in 99% (1496/1511), successfully avoiding downstream testing. In this cohort, a sensitivity analysis was conducted with an older population aged from 65 to 75 yr, and using cutoffs of Stockholm3 ≥ 11 and ≥ 15, the number of men referred for MRI and/or a biopsy using Stockholm3 compared with PSA ≥ 3 ng/ml could be reduced by 35% and 50%, respectively (Supplementary Tables 3 and 4).

Table 2 presents data for men with PSA ≥ 3 ng/ml and Stockholm ≥ 15, where 266 (2%) had PSA between 10 and 19.9 ng/ml. Of these men, 191 (72%) had an increased risk on Stockholm3 and underwent MRI, of whom 93 (35%) had PI-RADS ≥ 3 and underwent a biopsy. Of the biopsied men, 65 (67% of biopsied men and 24% of the tested men) were diagnosed with clinically significant prostate cancer. Table 3 shows data for men with PSA between 1.5 and 2.9 ng/ml and Stockholm3 ≥ 15. Of 2980 men with PSA 1.5–2.9 ng/ml, 360 (12%) had an increased risk on Stockholm3. These men underwent MRI, and 126 (35%) having positive MRI results were biopsied, of whom 72 (66%) were diagnosed with clinically significant prostate cancer.

**Table 2:** Men tested in 2018–2022 with PSA ≥ 3 ng/ml and Stockholm3 ≥ 15, and their MRI and biopsy outcomes divided into PSA categories

	All men with PSA ≥ 3 ng/ml	PSA (ng/ml)		
		3–9.9	10–19.9	≥20
Number of men	3496	3166	266	64
Men with positive Stockholm3, *n* (% of total)	1638 (47)	1395 (44)	191 (72)	52 (81)
Men undergoing MRI, *n* (% of total)	1612 (46)	1376 (43)	187 (70)	49 (77)
Positive MRI, *n* (% of total)	654 (19)	517 (16)	93 (35)	44 (69)
Number of biopsied men, *n* (% of total) a	680 (19)	536 (17)	97 (36)	47 (73)
Benign, *n* (% of total biopsied)	168 (25)	138 (26)	22 (23)	8 (17)
ISUP 1, *n* (% of total biopsied)	73 (11)	59 (11)	10 (10)	4 (9)
ISUP 2, *n* (% of total biopsied)	286 (42)	244 (46)	30 (31)	12 (26)
ISUP ≥ 3, *n* (% of total biopsied)	153 (22)	95 (18)	35 (36)	23 (49)

ISUP = International Society of Urological Pathology; MRI = magnetic resonance imaging; PSA = prostate-specific antigen.

a Not all men were able to undergo MRI and were therefore sent directly to biopsy.

**Table 3:** Men tested in 2018–2022 with PSA 1.5–2.9 ng/ml and Stockholm3 ≥ 15, and their MRI and biopsy outcomes

	All men with PSA 1.5–2.9 ng/ml	PSA (ng/ml)		
		1.5–1.9	2.0–2.4	2.5–2.9
Number of men	2980	1211	988	781
Men with positive Stockholm3, *n* (% of total)	360 (12)	51 (4)	131 (13)	178 (23)
Men undergoing MRI, *n* (% of total)	357 (12)	33 (3)	90 (9)	175 (22)
Positive MRI, *n* (% of total)	126 (4)	18 (1)	41 (4)	67 (9)
Number of biopsied men a,*n* (% of total)	129 (4)	18 (1)	41 (4)	70 (9)
Benign, *n* (% of total biopsied)	43 (33)	7 (39)	15 (39)	20 (29)
ISUP 1, *n* (% of total biopsied)	14 (11)	5 (28)	4 (10)	5 (7)
ISUP 2, *n* (% of total biopsied)	57 (44)	6 (33)	17 (41)	34 (49)
ISUP ≥ 3, *n* (% of total biopsied)	15 (12)	0 (0)	4 (10)	11 (16)

ISUP = International Society of Urological Pathology; MRI = magnetic resonance imaging; PSA = prostate-specific antigen.

a Not all men were able to undergo MRI and were therefore sent directly to biopsy.

**Fig. 3:**
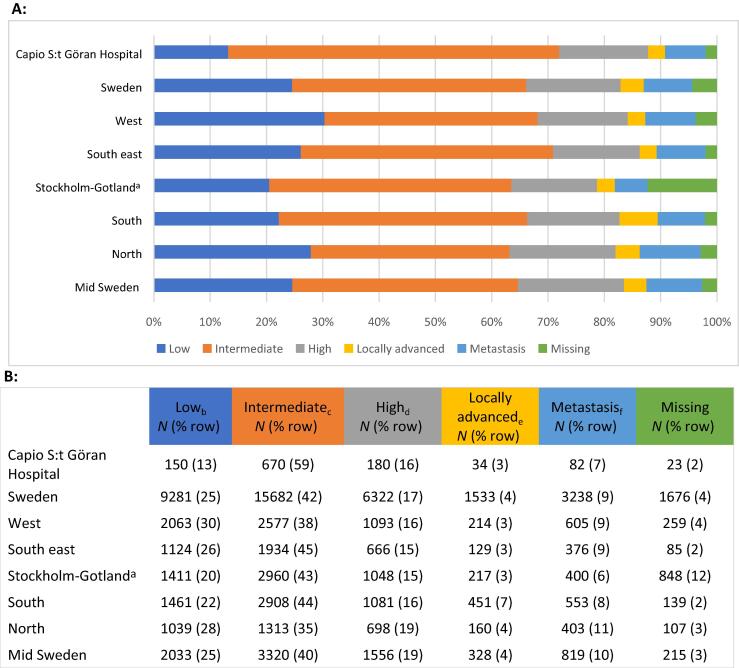
Regional variance in prostate cancer risk for men aged 45–75 yr diagnosed with prostate cancer during 2018–2022 in Sweden. Data were collected from the Swedish National Prostate Cancer Registry (NPCR) [17]. (A) Proportion of men in each risk category in Sweden and broken down into hospital regions. For comparison, we show the proportion of men in same risk categories of prostate cancer at Capio PCC. (B) Number of men and proportions in each risk category in Sweden and broken down into hospital regions. For comparison, we show the number of men in same risk categories diagnosed at Capio PCC. Capio PCC = Capio Prostate Cancer Center; ISUP = International Society of Urological Pathology; PSA = prostate-specific antigen. Risk group definition is from the NPCR in Sweden and national data extracted from NPCR’s interactive webpage. ^a^ Without the inclusion of Capio S:t Göran Hospital. ^b^ Low risk: T stage 1 or 2, ISUP grade 1, and PSA < 10 ng/ml. ^c^ Intermediate risk: T stage 1 or 2, ISUP grade 2–3, and/or PSA 10–19.9 ng/ml. ^d^ High risk: T stage 3 and/or ISUP 4–5 and/or PSA 20–49.9 ng/ml. ^e^ Locally advanced: T stage 4 and/or N stage 1, and/or PSA 50–99.9 ng/ml. ^f^ Metastasis: M stage 1 and/or PSA ≥ 100 ng/ml.

Figure 3 shows the national and regional data of prostate cancer risk in Sweden for men diagnosed with prostate cancer, aged 45–75 yr from 2018 to 2022, compared with men who were diagnosed with prostate cancer at Capio PCC. Capio PCC had fewer low-risk diagnoses (13% vs 25% in Sweden) and more intermediate-risk diagnoses (59% vs 42% in Sweden). Additionally, 26% of Capio PCC diagnoses were high risk, locally advanced, or metastatic compared with 30% in Sweden.

A cost analysis indicated that implementation of the Capio PCC model could reduce direct healthcare costs by approximately 25% compared with traditional diagnostics in Sweden, primarily due to fewer primary care and urologist visits and fewer unnecessary MRI scans and biopsies. See the Supplementary material for detailed cost assumptions and analysis.

## 4 Discussion

The Capio PCC model was designed with the dual purpose of enhancing the efficiency of prostate cancer testing and streamlining the diagnostic process for prostate cancer. By incorporating a blood-based diagnostic test and MRI, followed by targeted and systematic biopsies, the model aims to improve the accuracy and effectiveness of prostate cancer diagnostics. The real-world evidence from Capio PCC show that improved risk stratification of patients, contemporary imaging, and biopsy techniques as well as improved patient care decrease the number of low-risk prostate cancers by 48% and the number of MRI scans by 43%.

In our analysis, we used a PSA threshold of ≥ 3 ng/ml as a standard indication to MRI, in line with previously reported screening studies using PSA [2], [7], [18]. While some previous studies employ the standard biopsy method, Capio PCC uses a combined standard and targeted biopsy approach, making a direct biopsy outcome comparison challenging in our cohort. Nevertheless, a comparison can be made concerning the number of men referred for MRI, as PSA ≥ 3 ng/ml serves as a commonly used indication and is recommended as the primary screening test for prostate cancer by both the European Association of Urology and the American Urological Association [19], [20]. The Capio PCC model demonstrated a notable 43% reduction in MRI indications compared with PSA ≥ 3 ng/ml when using Stockholm3 ≥ 15, showcasing its efficacy in optimizing prostate cancer diagnostics.

In addition to Stockholm3, various tests aim to improve risk stratification and identify men at an increased risk of prostate cancer. These include blood-based assessments such as the Prostate Health Index and the 4KScore test, as well as urine-based examinations such as the prostate cancer antigen 3 test and the ExoDx Prostate IntelliScore. Additionally, risk calculators such as those from the European Randomized Study of Screening for Prostate Cancer and Prostate Biopsy Collaborative Group are relevant [21], [22], [23], [24], [25]. While clinical studies indicate promising values of these tests and risk calculators, particularly combined with MRI, the evidence on calibration and clinical net benefits remains controversial [26], [27], [28], [29]. Many of these tests or risk calculators need recalibration to demonstrate positive clinical net benefits, potentially impacting their clinical utility negatively.

The results from Capio PCC highlight the significance of detecting cancer in men with PSA between 1.5 and 2.9, as their cancer might otherwise go undetected due to the absence of symptoms. Thompson et al [30] demonstrated that cancers were not uncommon and generally found within the normal range in men with PSA levels below 4 ng/ml. In our cohort, men with PSA levels of ≥ 1.5 ng/ml were tested with Stockholm3, and those with an increased Stockholm3 risk were sent for MRI. Remarkably, 72 men (56% of those biopsied) with PSA levels between 1.5 and 2.9 ng/ml were found to have clinically significant prostate cancer. Moreover, our findings indicate that among men with PSA levels ranging from 10 to 19.9, employing the Stockholm3 test as a criterion for undergoing MRI scans could lead to a 28% reduction in the need for MRI in this risk group of men. Similarly, utilizing the Capio PCC model could result in a 63% decrease in the requirement for biopsies compared with using solely a PSA threshold of ≥ 3 ng/ml as a basis for biopsy recommendation.

The streamlined diagnostic process is a significant aspect of the Capio PCC model, benefiting both hospital staff and patients. By delegating tasks from specialist doctors to specially trained assistant nurses, the patient lead time has decreased substantially, resulting in shorter lead times than in other hospitals in Sweden [6]. Implementation of the Capio PCC model in other institutions outside Sweden requires access to the Stockholm3 test, MRI, and organizational willingness for task shifting.

Viste et al [31] and Bergman et al [5] have previously shown cost savings of 17% and 23–28%, respectively, using Stockholm3 compared with clinical practice with PSA. In addition, Hao et al [32] have shown cost effectiveness of Stockholm3 compared with PSA in a screening by invitation setting. In the current analysis, we estimated cost savings of 25% compared with a traditional diagnostic pathway in Sweden. There are limitations to this cost saving analysis as it is based only on Swedish data and cost assumptions. A more precise health economic analysis is needed for different clinical pathways and geographical areas.

Although this is one of the largest cohorts evaluating real-world data utilizing novel blood biomarkers and MRI for prostate cancer diagnosis, there are some limitations. Currently, the cancer status of the men with a low or normal risk on Stockholm3 or negative MRI results is unknown since these men were not biopsied. They will be retested in 2–6 yr depending on test result (men with a low risk on Stockholm3 will be called back for testing in 6 yr and men with normal risk or a negative MRI result will be retested in 2 yr). Preliminary results from these data show that both Stockholm3 and MRI have a high negative predictive value within a 2-yr timeframe, and very few cancers were detected in men with a normal risk on Stockholm3 or negative MRI in the first round. Furthermore, an exact count of men undergoing MRI procedures at Capio is unattainable due to the availability of data solely for those with positive MRI results. Nevertheless, our estimation relies on historical MRI data from Capio, which is expected to align closely with the actual figures.

Another limitation is in the health economic analysis where costs assumptions are specific to Swedish healthcare, which may not be generalizable to other healthcare systems in other countries. Work on studies including a more detailed health economic analysis has already started.

## 5 Conclusions

In conclusion, the diagnostic process at Capio PCC has shown advancements in prostate cancer detection and care. By utilizing a multivariable risk prediction tool alongside MRI scans and a combination of systematic and targeted biopsies, there have been notable decreases in the use of MRI and a reduction in identifying low-risk cancers compared with conventional prostate cancer diagnostic approaches. This approach has led to enhancements in the overall accuracy and effectiveness of prostate cancer diagnosis and treatment.

  ***Author contributions*:** Thorgerdur Palsdottir had full access to all the data in the study and takes responsibility for the integrity of the data and the accuracy of the data analysis.

*Study concept and design*: Palsdottir, Söderbäck, Jäderling, Bergman, Vigneswaran, Grönberg.

*Acquisition of data*: Söderbäck, Jäderling, Bergman, Grönberg.

*Analysis and interpretation of data*: Palsdottir, Vigneswaran, Grönberg.

*Drafting of the manuscript*: Palsdottir.

*Critical revision of the manuscript for important intellectual content*: Palsdottir, Söderbäck, Jäderling, Grönberg.

*Statistical analysis*: Palsdottir.

*Obtaining funding*: Grönberg.

*Administrative, technical, or material support*: None.

*Supervision*: Grönberg.

*Other*: None.

  ***Financial disclosures:*** Thorgerdur Palsdottir certifies that all conflicts of interest, including specific financial interests and relationships and affiliations relevant to the subject matter or materials discussed in the manuscript (eg, employment/affiliation, grants or funding, consultancies, honoraria, stock ownership or options, expert testimony, royalties, or patents filed, received, or pending), are the following: Henrik Grönberg reports five pending prostate cancer diagnostic–related patents: Method for indicating a presence or non-presence of aggressive prostate cancer (WO0213EP74259201311120); Prognostic method for individuals with prostate cancer (WO2013EP7427020131120); Method for indicating a presence of prostate cancer in individuals with particular characteristics (WO2018EP5247320180201); Method for indicating the presence or non-presence of prostate cancer (WO2013SE5055420130516); and Method for detecting solid tumour cancer (WO2015SE5027220150311). The Karolinska Institutet collaborates with A3P Biomedical in developing the technology for the Stockholm3 test. Henrik Grönberg reports owning shares in A3P Biomedical, and Thorgerdur Palsdottir and Hari Vigneswaran are partially employed by A3P Biomedical. All other authors declare no competing interests.

  ***Funding/Support and role of the sponsor:*** This work was supported by the Swedish Cancer Society, the Swedish Research Council.
