# Supplementary material for: The Capio Prostate Cancer Center Model for Prostate Cancer Diagnostics—Real-world Evidence from 2018 to 2022
Source: Eur Urol Open Sci. 2024 Feb 6;61:29–36. doi: 10.1016/j.euros.2024.01.012 (PMC10879938; doi:10.1016/j.euros.2024.01.012)
Supplement: Supplementary data 1 [file mmc1.docx]

**Supplementary Tables and Figures**

**Supplementary Figure 1:** Standardised protocoll driven diagnostic chain at Capio PCC vs. standard primary care outpatient urology process.

*****Normal process, process for individual patients can vary.

**Supplementary Figure 2:** Consort diagram 2018-2022

**Supplementary Figure 3:** Number of Stockholm3 tests per year at Capio PCC from 2018-2022.


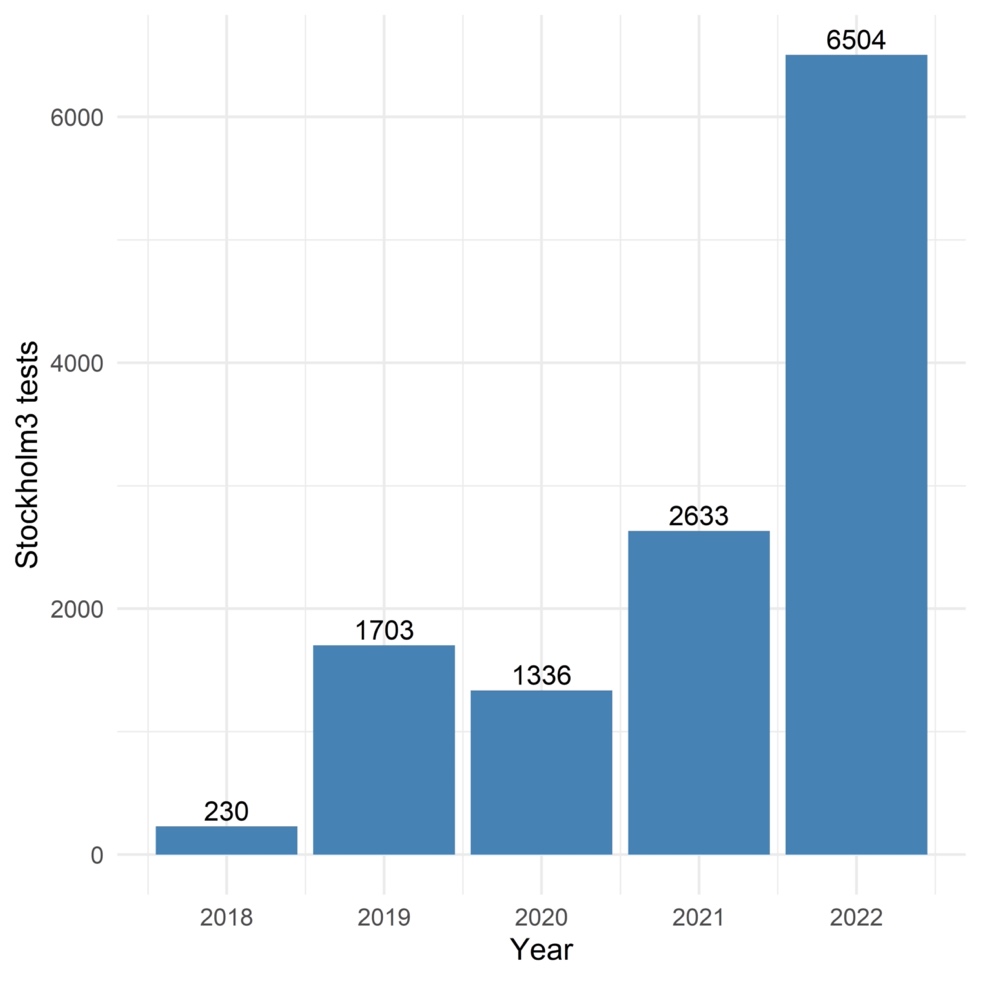


**Supplementary Figure 4:** Health economic analysis

Notes:

1. Observed proportion of men tested at Capio PCC.

2. Share positive MRI (PIRADS ≥ 3) if only PSA is used. Data based on Gothenburg-2 study [18].

3. Share positive MRI (PIRADS ≥ 3) if PSA is followed by Stockholm3 as reflex.

4. Share men with high-risk prostate cancer in biopsy.

5. Team analysis. Monthly salary doctor x taxes and pension x Overhead / (number of patients/day x 20 day/month) = EUR 8,000 x 1.5 x 1.3 / (6 x 20) = EUR 130/visit.

6. Estimation based on price list Region Stockholm for in-hospital urologist visit.

7. Team analysis. Monthly salary sub nurse x taxes & pension x Overhead / (number of patients/day x 20 day/month) = EUR 3,000 x 1.5 x 1.3 / (20 x 20) ≈ EUR 15/patient.

8. Estimation based on price list Karolinska University Hospital including sampling costs.

9. Price list A3P Biomedical.

10. Estimating based on price quote from private radiology in Stockholm.

11. Estimation based on Region Stockholm price list for urology and price list Karolinska University Laboratory for handling and pathology evaluation of prostate biopsy.

12. Price list Region Stockholm for multi-disciplinary conference for high-risk cancer.

**Supplementary Table 1:** Men tested 2018 to 2021 with PSA <3 ng/ml and Stockholm3 ≥11 and their MRI and biopsy outcomes.

|  | **All men with PSA 1.5 – 2.9 ng/ml** | **PSA (ng/ml)** | | |
| --- | --- | --- | --- | --- |
|  |  | **1.5 – 1.9** | **2.0 – 2.4** | **2.5 to 2.9** |
| Number of men | 1292 | 483 | 449 | 360 |
| Men with a positive Stockholm3  (n, % of total) | 354 (27) | 75 (16) | 126 (28) | 153 (42) |
| Men undergoing MRI  (n, % of total) | 351 (27) | 74 (15) | 82 (18) | 151 (42) |
| Positive MRI (n, % of total) | 140 (11) | 38 (8) | 44 (10) | 58 (16) |
| Number of biopsied men  (n, % of total) | 143 (11) | 39 (8) | 44 (10) | 60 (17) |
| Benign  (n, % of total biopsied) | 51 (36) | 12 (31) | 21 (48) | 18 (30) |
| ISUP 1 (n, % of total biopsied) | 21 (15) | 8 (21) | 6 (14) | 7 (12) |
| ISUP 2 (n, % of total biopsied) | 61 (43) | 18 (46) | 13 (30) | 30 (50) |
| ISUP ≥3 (n, % of total biopsied) | 10 (7) | 1 (3) | 4 (9) | 5 (8) |

**Supplementary Table 2:** Men tested 2018 to 2021 with PSA <3 ng/ml and Stockholm3 ≥15 and their MRI and biopsy outcomes.

|  | **All men with 1.5 ≥ PSA < 3 ng/ml** | **PSA (ng/ml)** | | |
| --- | --- | --- | --- | --- |
|  |  | **1.5 – 1.9** | **2.0 – 2.4** | **2.5 to 2.9** |
| Number of men | 1292 | 483 | 449 | 360 |
| Men with a positive Stockholm3  (n, % of total) | 161 (12) | 24 (5) | 62 (14) | 75 (21) |
| Men undergoing MRI  (n, % of total) | 160 (12) | 11 (2) | 37 (8) | 74 (21) |
| Positive MRI (n, % of total) | 75 (6) | 13 (3) | 25 (6) | 37 (10) |
| Number of biopsied men  (n, % of total) | 76 (6) | 13 (3) | 25 (6) | 38 (11) |
| Benign  (n, % of total biopsied) | 26 (34) | 3 (23) | 13 (52) | 10 (26) |
| ISUP 1 (n, % of total biopsied) | 10 (13) | 4 (31) | 3 (12) | 3 (8) |
| ISUP 2 (n, % of total biopsied) | 33 (43) | 6 (46) | 7 (28) | 20 (53) |
| ISUP ≥3 (n, % of total biopsied) | 7 (9) | 0 (0) | 2 (8) | 5 (13) |

**Supplementary Table 3:** Men tested 2018 to 2021 aged 65-75 years with Stockholm3 ≥11 and their MRI and biopsy outcomes.

|  | **Men age ≥65 yrs with PSA ≥1.5 ng/ml** | **PSA (ng/ml)** | | | |
| --- | --- | --- | --- | --- | --- |
|  |  | **1.5 – 2.9** | **3.0 – 9.9** | **10-19.9** | **≥20** |
| Number of men | 1570 | 545 | 886 | 109 | 30 |
| Men with a positive Stockholm3  (n, % of total) | 882(56) | 190(35) | 578(65) | 89(82) | 25(83) |
| Men undergoing MRI  (n, % of total) | 861(55) | 187(34) | 563(64) | 87(80) | 24(80) |
| Positive MRI (n, % of total) | 386(25) | 79(14) | 245(28) | 42(39) | 20(67) |
| Number of biopsied men  (n, % of total) | 407(26) | 82(15) | 260(29) | 44(40) | 21(70) |
| Benign  (n, % of total biopsied) | 127(31) | 36(44) | 80(31) | 8(18) | 3(14) |
| ISUP 1 (n, % of total biopsied) | 42(10) | 11(13) | 27(10) | 2(5) | 2(10) |
| ISUP 2 (n, % of total biopsied) | 166(41) | 32(39) | 110(42) | 15(34) | 9(43) |
| ISUP ≥3 (n, % of total biopsied) | 72(18) | 3(4) | 43(17) | 19(43) | 7(33) |

**Supplementary Table 4:** Men tested 2018 to 2022 aged 65-75 years with Stockholm3 ≥15 and their MRI and biopsy outcomes.

|  | **Men age ≥65 yrs with PSA ≥1.5 ng/ml** | **PSA (ng/ml)** | | | |
| --- | --- | --- | --- | --- | --- |
|  |  | **1.5 – 2.9** | **3.0 – 9.9** | **10-19.9** | **≥20** |
| Number of men | 2994 | 1203 | 1587 | 167 | 37 |
| Men with a positive Stockholm3  (n, % of total) | 1175(39) | 233(19) | 790(50) | 121(72) | 31(84) |
| Men undergoing MRI  (n, % of total) | 1156(39) | 230(19) | 778(49) | 119(71) | 29(78) |
| Positive MRI (n, % of total) | 486(16) | 83(7) | 314(20) | 64(38) | 25(68) |
| Number of biopsied men  (n, % of total) | 505(17) | 86(7) | 326(21) | 66(40) | 27(73) |
| Benign  (n, % of total biopsied) | 134(27) | 33(38) | 83(25) | 14(21) | 4(15) |
| ISUP 1 (n, % of total biopsied) | 54(11) | 9(10) | 39(12) | 4(6) | 2(7) |
| ISUP 2 (n, % of total biopsied) | 208(41) | 37(43) | 140(43) | 22(33) | 9(33) |
| ISUP ≥3 (n, % of total biopsied) | 109(22) | 7(8) | 64(20) | 26(39) | 12(44) |

**Supplementary material:**

**Diagnostic process at Capio PCC – The full Capio Model**

The diagnostic chain structure is as follows and is referred to as the Capio PCC Model text:

1. Men request prostate cancer testing on Capio PCC‘s secure website or they are referred from general practionioners. The man answer a questionnaire on the website, providing information on the clinical variables needed as input for the Stockholm3 as well as information to identify those who do not benefit from prostate cancer testing (men aged 45-75 years). This is done to reduce the problem of over-testing among yong and older men.
2. Following the online questionnaire, the man receives a referral to a clinic for blood-sampling with the Stockholm3 at one of many locations in Stockholm. In addition, the man will receive an information sheet to his home address on the pros and cons of prostate cancer testing from the Swedish National Board of Health and Welfare.
3. Stockholm3 is a reflex test performed if the PSA ≥ 1.5 ng/ml and gives the man a risk score for clinically significant prostate cancer (ISUP grade ≥2). As such, Stockholm3 is automatically performed if the PSA level is 1.5 ng/ml or higher without the patient having to provide additional blood samples.
4. The men are divided into three Stockholm3 risk categories, low, medium and increased prostate cancer risk. The men who are considered to be at increased risk for significant prostate cancer are contacted by a specially trained assistant nurse by phone to provide the test result and book a time within 1-2 weeks for an MRI of the prostate. The men are asked if they have a hip-replacement and these men are booked for a longer MRI session for the possibility to add DCE in cases where DWI is not diagnostic due to severe artifacts.
5. The man will then undergo a 16-minute MRI without dynamic contrast enhancement (DCE) containing T2-weighted, T1-weighted and functional diffusion-weighted images (DWI). The MRI findings are classified according to the Prostate Imaging – Reporting and Data System (PI-RADS) v.2.1 [15].
6. The men with cancer suspicious lesions (PI-RADS ≥3) are again contacted by an assistant nurse to inform the man of the MRI results and book an appointment with a urologist for a prostate biopsy. That is the first visit to the urologist in the diagnostic chain. Men with PI-RADS 3 undergo targeted biopsies (3-5 biopsy cores) per lesion while men with PI-RADS 4-5 undergo a combination of targeted and systematic biopsies (10-12 cores). Only performing targeted biopsies in men with PI-RADS 3 lesions is done to reduce the risk of overdiagnosis of low-risk cancer. At the prostate cancer center, biopsies have also been performed in men with low Stockholm3 risk associated with a positive digital rectal examination (DRE).
7. Within 1-2 weeks a pathology evaluation is reported and a multidisciplinary conference (MDC) is held within the group of health professionals at the center to discuss the biopsy results, and to plan and coordinate the treatment of the patient.
8. When biopsy result is ascertained, the responsible urologist will contact the man directly to report the biopsy result and for those with a positive result for cancer, a visit to the urologist is arranged.
9. Men with a low risk on Stockholm3 return for retesting in 6 years and men with normal and increased risk on Stockholm3 with negative MRI are recommended to return for retesting in 2 years.

**Questionnaire for referral to blood testing at Capio PCC**
